# Plant polyphenols can protect VEGF-induced vascular leakage?

**DOI:** 10.17179/excli2016-128

**Published:** 2016-02-18

**Authors:** Cijo George Vazhappilly

**Affiliations:** 1 Department of Environmental Sciences, Faculty of Agriculture, Dalhousie University, Truro, Canada

## Dear Editor,

The vascular system performs the critical functions of supplying tissues with nutrients and clearing waste products. Vascular leakage, caused by acute or chronic exposure to vascular endothelial growth factor (VEGF), is unique amongst angiogenic factors since it primarily results in endothelial dysfunction/disruption (Bao et al., 2009[1]). VEGF was initially described as a vascular permeability factor produced by some tumors, or during ischemia, since it was a potent inducer of vascular leakage and edema. This may result in the invasion of tumor cells, intravasation into circulation, extravasation at distant sites, and permeability to solute and proteins at physiological and pathological conditions. VEGF disrupts the endothelial barrier and breaks cell-cell contact by inhibiting tight junctional proteins and activating ROS-dependent Src kinases (Gavard, 2009[2]). Phosphorylation of different proteins in the Src family will results in leakage, which in turn can result in severe conditions like metastatic cancer, edema, hemophilia, atherosclerosis, etc. So it is crucial to investigate the molecular mechanisms of vascular leakage of endothelial cells. 

Recent research advancements have brought new insights into the regulation of these proteins by various agents including exogenous expression of FVIIa or silver nanoparticles in order to decrease VEGF-induced vascular leakage (Sundaram et al., 2014[6]; Sheikpranbabu et al., 2009[5]) though, its mechanism of action is not completely understood. However, there is a lack of knowledge in how the dietary suppliants, which are rich source of antioxidants, regulate these mechanisms in human body. Medicinal plants are considered to be the main sources of biologically active compounds, especially flavonoids that can be used for the treatment of various ailments including cancer, coronary atherosclerosis and various blood disorders (Naveen et al., 2013[4]). 

Polyphenols including luteolin, curcumin and epigalloccatechin-3-gallate (EGCG) have been known to inhibit angiogenesis or tumor neovasculature formation, but the underlying mechanisms have not been well studied. Resveratrol, a polyphenolic compound found in grapes and other fruits has been reported to inhibit VEGF-induced vascular leakage in endothelial cells through the Src pathway (Lin et al., 2003[3]). Nevertheless, there is a dearth of knowledge regarding the potential of major flavonoids, and their mode of action in inhibiting VEGF-induced leakage in vascular endothelium. Extensive studies on their effectiveness are essential, since dietary-rich polyphenols can inhibit tumor metastasis and help to restore hemostasis.

## Conflict of interest

The author declares that he has no conflict of interest. 
